# What triggers colour change? Effects of background colour and temperature on the development of an alpine grasshopper

**DOI:** 10.1186/s12862-015-0419-9

**Published:** 2015-08-21

**Authors:** J. Pablo Valverde, Holger Schielzeth

**Affiliations:** Department of Evolutionary Biology, Bielefeld University, Morgenbreede 45, 33615, Bielefeld, Germany

**Keywords:** Acrididae, Crypsis, Developmental plasticity, Environmental predictability, Homochromy, Orthoptera, Phenotypic plasticity, Thermoregulation

## Abstract

**Background:**

Colour polymorphisms are a fascinating facet of many natural populations of plants and animals, and the selective processes that maintain such variation are as relevant as the processes which promote their development. Orthoptera, the insect group that encompasses grasshoppers and bush crickets, includes a particularly large number of species that are colour polymorphic with a marked green-brown polymorphism being particularly widespread. Colour polymorphism has been associated with the need for crypsis and background matching and background-dependent homochromy has been described in a few species. However, when and how different environmental conditions influence variation in colour remains poorly understood. Here we test for effects of background colour and ambient temperature on the occurrence of colour morph switches (green to brown or brown to green) and developmental darkening in the alpine dwelling club-legged grasshopper *Gomphocerus sibiricus*.

**Results:**

We monitored individually housed nymphae across three of their four developmental stages and into the first week after final ecdysis. Our data show an absence of colour morph switches in *G. sibiricus*, without a single switch observed in our sample. Furthermore, we test for an effect of temperature on colouration by manipulating radiant heat, a limiting factor in alpine habitats. Radiant heat had a significant effect on developmental darkening: individuals under low radiant heat tended to darken, while individuals under high radiant heat tended to lighten within nymphal stages. Young imagoes darkened under either condition.

**Conclusions:**

Our results indicate a plastic response to a variable temperature and indicate that melanin, a multipurpose pigment responsible for dark colouration and presumed to be costly, seems to be strategically allocated according to the current environmental conditions. Unlike other orthopterans, the species is apparently unable to switch colour morphs (green/brown) during development, suggesting that colour morphs are determined genetically (or very early during development) and that other processes have to contribute to crypsis and homochromy in this species.

## Background

Colour polymorphism has fascinated biologists since the time of Darwin, and its evolutionary meaning is still being revealed [1–3]. Colour polymorphism, defined here as within-species phenotypic variation, occurs throughout the animal kingdom in several taxa of birds, fish, mammals, frogs, molluscs, spiders, several insect orders and also in plants [4–9]. The occurrence of colour polymorphisms in natural populations can result from biased mutation, pleiotropy and trade-offs, gene flow, spatially and/or temporally fluctuating selection and negative frequency-dependent selection that can counter loss of variation by genetic drift [10–13]. Furthermore, developmental plasticity and phenotypic flexibility, if they do not invoke significant cost, might allow the maintenance of polymorphisms. This can be particularly advantageous in unpredictably variable environments.

Insects offer a multitude of examples for the coexistence of two or more colour morphs in groups such as grasshoppers, mantoids, cicadids, damselflies, lepidopterans and beetles [13–15]. There is ample evidence for genetic and environmental effects, as well as genotype-by-environment interactions in colour determination [14–18]. Several species appear capable of modifying their colour in response to various environmental cues such as temperature, predation threats, behaviour stimulus (e.g., crab spiders which try to blend with their background to ambush prey, [6]), among others [19]. Within Orthoptera, colour polymorphism is present in dozens of species (reviewed in [15], see also [19, 20]). Two particularly eye-catching forms of colour polymorphism in orthopterans are a widespread green-brown polymorphism in grasshoppers and bush crickets and the famous phase polymorphism in locusts [14, 21]. Phase polymorphism is triggered by changes in population density which induces changes in colour (typically black patterns in gregarious versus pale green or brown colours in solitary phases) as part of more complex changes in morphology, physiology, behaviour and life history [22–26].

The green-brown polymorphism is far more widespread among orthopterans than phase polymorphism and does not correlate with obvious changes in morphology and/or behaviour. Many families and genera of orthopterans comprise species that display either green or brown morphs, while other species are polymorphic (e.g., in genera *Decticus*, *Metrioptera*, *Oedaleus*). In some species, one of the morphs is very rare (such as brown morphs in *Decticus verrucivorus*), while in others the ratios are far more even (as in *Metrioptera roeselii*) [14]. With respect to environmental effects, green morphs seem to develop primarily under high humidity, while brown morphs are favoured under dry environmental conditions [15, 27, 28]. Besides the two very striking forms of colour polymorphism mentioned before, there is a range of more fine-scaled within-species variation in colour pattern and colouration among orthopterans [29, 30]. Groundhoppers, for example, differ substantially in their colour patterns, which can be categorized into variable numbers of discrete morphs [31, 32]. In other species, differences in colour are more continuous such as with colouration of species in the genus *Oedipoda*. Such fine-scaled variation seems to be partly under genetic, partly under environmental control [15, 16, 33]. Many species also show occasional pinkish, purple, yellow or blue colour morphs [34], further illustrating the diversity of colour in orthopterans.

A very interesting phenomenon associated with colour polymorphism is homochromy, which describes matching of body colouration with variation in the background pattern of the local habitat [14, 15, 20, 35]. Such matching might arise for four different reasons: (i) local adaptation due to multi-generational history of selection on genetic polymorphisms, (ii) selective mortality within generations, (iii) individual-level choice of matching habitat patches [36], and (iv) developmental plasticity of body colouration to match local conditions [14]. Developmental switches are particularly intriguing, because they allow individual-level matching, which is likely advantageous if habitats are unpredictably variable across generations, but predictable from environmental cues over the lifetime of individuals. Developmental matching has been reported in orthopterans for species with fine-scale variation in colour pattern [37, 38], but also for species which present the green and brown colour polymorphism (Table 1).

**Table 1:** Studies on the effects of background colouration on the occurrence of colour morph switches in green-brown polymorphic (upper section) and other polymorphic (lower section) orthopterans

		*N*		Proportion of Switches			
Species	Background Material	matched	non-matched	matched	non-matched	Stages with Switches	Reference
a) Green-Brown Polymorphism							
*Acrida turrita*	fresh/dry grass	62	145	4 %	86 %	after ecdysis	[48]
*Acrida turrita*	painted sawdust	14	15	0 %	100 %	after ecdysis	[48]
*Acrida turrita*	painted sawdust		32		0 %	imago	[48]
*Oedaleus decorus*	NA	10	66	0 %	88 %	after ecdysis	[33]
*Schistocerca americana*	coloured paper		74		0 %	across stages	[47]
*Schistocerca gregaria*	coloured paper	58	312	0 %	63.5 %	within stages	[68]
*Locusta migratoria*	coloured paper	58	237	69 %	24 %	across stages	[69]
*Rhammatocerus*	habitat background		3000 >		90 % >	imago	[28]
*schistocercoides*							
*Chorthippus biggutulus*	coloured paper		257		30 %	across stages	[70]
b) Other Polymorphisms							
*Oedipoda sp.*	earth, clay, coal, stone, chalk	12	92	0 %	80 %	after ecdysis	[37]
*Tetrix subulata*	sand		312		16 %	across stages	[49]
*Tetrix ceperoi*	sand		228		16 %	across stages	[49]

The proportion of switches was calculated for various studies based on multiple assays either on matched or non-matched background colour. Studies do not indicate precise time of colour morph switch occurrence, only final results of repeated colour assessments across nymphal stages are stated (in the case of Ergene all switches occur after an ecdysis event). Percentages reflect amount of individuals from the total amount in any given category – matched or non-matched – which switched colour, therefore they are not expected to add up to 100 %. Most studies used low densities of individuals (≤5) per cage, but one study ([33]) housed up to 10 individual per cage. NA = information not available

Orthopterans are preyed upon by a large diversity of species, including birds, lizards, amphibians, spiders and other insects and are frequently parasitized by parasitic flies and mites [24, 34]. Visually hunting predators might constitute a force that can favour homochromy and crypsis, since survival to the imago stage is critical to individual fitness. Predators might also impose frequency-dependent selection if they develop search images and preferentially prey upon the most common morphs [9]. However, there are other influences that might affect body colour and this may or may not be in conflict with crypsis. For example, body colour is likely to affect the absorption of radiant heat and therefore play an important role in thermoregulation [13, 39–41]. It has repeatedly been reported that orthopterans raised under cool conditions are darker than those raised under warm conditions [16, 19, 42–44].

The club-legged grasshopper *Gomphocerus sibiricus* is a highly sexually dimorphic alpine dwelling grasshopper that exhibits the green-brown polymorphism present in many other orthopterans. Green individuals are rarer than brown morphs in most populations. Despite substantial fluctuations in population density [24, 45], the species does not show any typical phase polymorphism [26]. It inhabits alpine pastures and grassland with a very heterogeneous composition of open terrain strewn with stones and mottled by various types of short grasses and herbaceous plants. Climate conditions in the mountains are very unpredictable and variable within and between years. The maintenance of the green-brown colour polymorphism could be aided by the heterogeneous habitat and/or temporal variability in climate conditions in the native habitat of *G. sibiricus*.

In the present study we aimed to test the effect of two known factors on developmental colour changes in *G. sibiricus*. First, we assessed the effect of background colour (green or brown) on colour morph development across almost the entire ontogeny. We were particularly interested in whether individuals are able to switch colour morphs to achieve homochromy as it has been described in other species (Table 1). We predicted that if individuals were able to switch colour morphs, then individuals whose colour morph mismatched the background colour would be capable of matching their background at an advanced developmental stage. Second, we assessed the effect of temperature by means of a radiant heat treatment on developmental darkness, while controlling for humidity, population density and food moisture content. Here we predicted that if individuals were capable of manipulate the degree of melanin in their cuticle, thermoregulation needs would promote a colouration darkening under conditions of low radiation. We followed individuals from the second nymphal stage through to the imaginal stage during two independent rounds of trials with two different radiant heat regimes. Individuals were exposed to experimental treatments from the second nymphal stage onwards. The long exposure to experimental conditions allowed us to evaluate if colour changes occur exclusively in connection with moults or if changes were possible even within nymphal stages.

## Results

A total of 78 individuals survived the first nymphal stage and entered the experimental setup in two rounds (referred to as R1 and R2). From this total, 34 individuals matched their cage background, while 44 did not match. Sixty one individuals were brown (78 %, 36 in R1 and 25 in R2) and 17 green (22 %, 6 in R1 and 11 in R2). The distribution of morphs in the R2 differed significantly between the sexes with brown individuals of both sexes (11 males = 44 %, 14 females = 56 %), but only green females (11 individuals) (*Χ*^2^ = 5.05, df = 1, p = 0.025).

### Colour morph switches

No colour morph switch was observed among the 34 individuals in cages with matched background colours (6 green individuals in green cages and 28 brown in brown cages in total for both rounds). Forty-four individuals (33 brown and 11 green) were exposed to unmatched backgrounds, but no colour morph switches were observed among these 44 individuals. Reasoning based on binomial sampling (see methods section) suggests that if *G. sibiricus* is capable of switch colour, the rate of colour morph switches is well below values reported in previous studies (*c* ≤ 0.07, Table 2). When we concentrate on the subset of the data where colour morph switches were most likely, given both non-matched background and temperature treatment (brown individuals on a green background under high radiant heat treatment), the probability of colour morph switch is still well below expectations (*c* ≤ 0.17, Table 2).

**Table 2:** Number of individuals of G. sibiricus on mismatched backgrounds, all of which did not switch colour morph during development

Round 1			
Morph & Background	Temperature		
	High	Low	Sum
Green on Brown	*n* = 3	*n* = 0	*n* = 3
	c ≤ 0.63	NA	c ≤ 0.63
Brown on Green	*n* = 9	*n* = 9	*n* = 18
	c ≤ 0.28	c ≤ 0.28	c ≤ 0.15
Sum	*n* = 12	*n* = 9	*n* = 21
	c ≤ 0.22	c ≤ 0.28	c ≤ 0.13
Round 2			
Morph & Background	Temperature		
	High	Low	Sum
Green on Brown	*n* = 4	*n* = 4	*n* = 8
	c ≤ 0.53	c ≤ 0.53	c ≤ 0.31
Brown on Green	*n* = 7	*n* = 8	*n* = 15
	c ≤ 0.35	c ≤ 0.31	c ≤ 0.18
Sum	*n* = 11	*n* = 12	*n* = 23
	c ≤ 0.24	c ≤ 0.22	c ≤ 0.12
Round 1 + 2			
Morph & Background	Temperature		
	High	Low	Sum
Green on Brown	*n* = 7	*n* = 4	*n* = 11
	c ≤ 0.35	c ≤ 0.53	c ≤ 0.24
Brown on Green	*n* = 16	*n* = 17	*n* = 33
	c ≤ 0.17	c ≤ 0.16	c ≤ 0.09
Sum	*n* = 23	*n* = 21	*n* = 44
	c ≤ 0.012	c ≤ 0.13	c ≤ 0.07

Table depicts number of individuals for each of two rounds under two sets of radiant heat regimes and values for the two combined rounds. The c value shows the range of colour switch probabilities that are consistent with the data at α = 0.05

### Temperature-cued colouration darkening

**Fig. 1:**
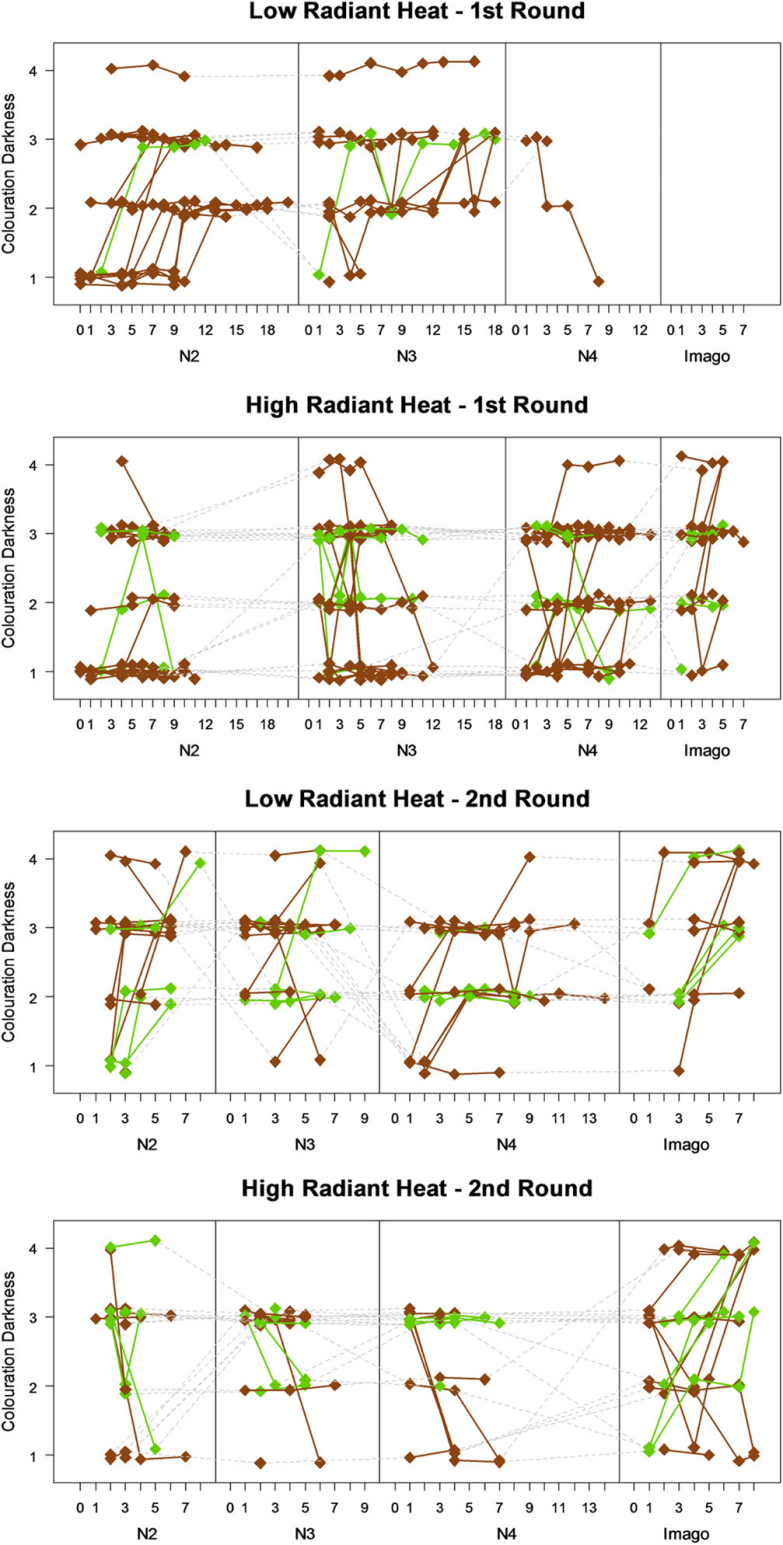
Changes in darkness across nymphal stages exposed to two different sets of radiant heat regimes (first round R1, and second round R2). In each set of panels, upper panels show low radiant heat treatments, bottom panels show high radiant heat treatments. Each line represents the trajectory of an individual (some individuals have only one observation per nymphal stage, and therefore appear only as a dot without line). Brown lines represent brown morphs, while green lines represent green morphs. Nymphal stages are measured in days since the start of each nymphal stage. Darkness has four levels, with 1 representing the lightest colour morph and 4 representing the darkest colour morph

We observed a significant change in colouration darkness in both rounds of trials. Individuals in the second and third nymphal stages (N2 and N3) of the round 1, and in the N2 and N4 stages of the round 2 experienced a change in darkness which depended on the direction of the temperature treatment. Individuals in the low radiant heat treatment became darker in colour, while those in the high radiant heat treatment became lighter in colour (Table 3, Fig. 1). Individuals in the N3 stage in the R2 did not show a significant change in colour, yet the sign of the point estimate is the same as in stages N2 and N4. Individuals in the N4 stage of the R1 and under a high radiant heat treatment got significantly darker with age. Most of the low radiant heat treatment individuals from the R1 had perished, with the single remaining individual becoming lighter. Unlike the situation in nymphal stages, individuals in the imago stage undergo a darkening in colour in both treatments within the first week after final ecdysis (Fig. 1).

**Table 3:** Results of the random-slope mixed effects model used to test for effects of radiant heat treatment and larval age on colouration darkness in both green and brown coloured morphs

	Round 1				Round 2			
	slope	SE	*t*	*p*	slope	SE	*t*	*p*
	N2 (*n* = 42)				N2 (*n* = 35)			
Intercept	1.79	0.25	7.16	<0.0001	1.92	0.33	5.77	<0.0001
High radiant heat treatment	0.28	0.34	0.82	0.42	1.02	0.52	1.98	0.057
Larval age	0.063	0.017	3.78	0.00054	0.18	0.061	2.92	0.0065
Treatment × Age	−0.067	0.027	−2.49	0.022	−0.38	0.12	−3.29	0.0025
	N3 (*n* = 34)				N3 (*n* = 34)			
Intercept	2.23	0.28	8.04	<0.0001	2.55	0.2	12.77	<0.0001
High radiant heat treatment	0.23	0.35	0.66	0.51	0.39	0.31	1.27	0.21
Larval age	0.036	0.014	2.65	0.013	0.035	0.045	0.79	0.43
Treatment × Age	−0.088	0.023	−3.82	0.00062	−0.12	0.076	−1.62	0.11
	N4 (*n* = 22)				N4 (*n* = 32)			
Intercept	2.03	0.19	10.62	<0.0001	1.85	0.2	9.06	<0.0001
High radiant heat treatment	NA	NA	NA	NA	1.04	0.3	3.42	0.0019
Larval age	0.033	0.015	2.21	0.039	0.078	0.034	2.26	0.032
Treatment × Age	NA	NA	NA	NA	−0.22	0.061	−3.54	0.0014
	IM (*n* = 21)				IM (*n* = 29)			
Intercept	NA	NA	NA	NA	1.88	0.36	5.21	<0.0001
High radiant heat treatment	NA	NA	NA	NA	0.34	0.45	0.76	0.45
Larval age	NA	NA	NA	NA	0.21	0.066	3.26	0.0032
Treatment × Age	NA	NA	NA	NA	−0.092	0.082	−1.13	0.27

Panels on the left and right side of the table show results for the first and second round, respectively. The number of individuals present in the experimental setup is depicted at the top of each block. The analysis of N4 in the first round only includes the test for the effect of age because almost all individuals in the low radiant heat treatment had died at the start of the nymphal stage. NA = not available

The analysis of unambiguous cases of lightening or darkening using Fisher’s exact tests confirmed a significant colour change for the N2 and N4 stages in the R2 (N2 stage: *n* = 14, *p* = 0.023; N3 stage: *n* = 8, *p* = 0.14; N4 stage: *n* = 10, *p* = 0.015; IM stage: *n* = 12, *p* = 0.47, see also Fig. 1). Yet after exclusion of ambiguous cases, there was not significant effect in the R1 (N2 stage: *n* = 12, *p* = 0.091; N3 stage: *n* = 7, *p* = 0.28; N4 stage: *n* = 12, *p* = 0.33, see also Fig. 1).

**Fig. 2:**
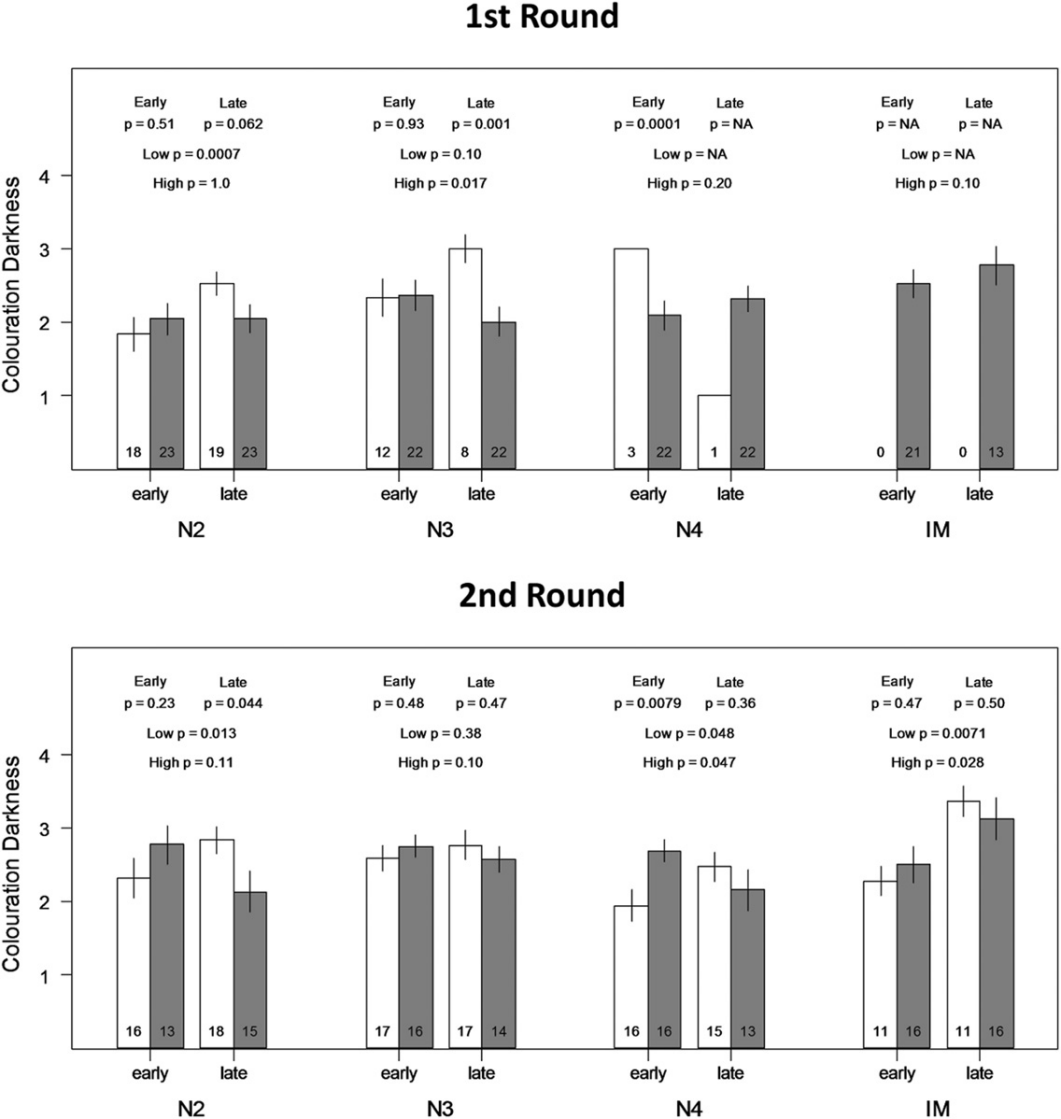
Mean darkness per nymphal stage at the earliest and latest observation point, separated per radiant heat treatment for two different sets of radiant heat regimes. Upper panel shows the first round (R1), lower panel shows the second round (R2). White bars represent low radiant heat treatments, grey bars represent high radiant heat treatments. Numbers inside the bar show the number of individuals per observation point. P-values for two-sample t-tests comparing treatments within observation points are shown on top (labeled as “Early” and “Late”). P-values for paired t-tests comparing observation points within treatments are shown below (labeled as “High” and “Low”)

The comparison of treatments per observation time (early or late observations within nymphal stages) also confirmed a significant effect of the treatment in the colour of nymphae (upper p values in Fig. 2). Individuals in the N2 stage in both rounds, and in the N3 stage of R1 show a significant difference in colouration darkness at the end of the nymphal stage. Individuals of the N4 stage differ at the start of the nymphal stage, but at the end colouration darkness had converged.

Finally the comparison between the earliest and latest observations per treatment within nymphal stages also found a significant effect of the treatment in the colouration darkness of nymphae (lower p values in Fig. 2). Individuals in the N2 stage of both rounds (statistically significant only in the low radiant heat treatment), N3 stage of R1 (statistically significant only in the high radiant heat treatment) and N4 stages of R2 change their colouration darkness within nymphal stages. The colour change in the IM stage is also significant and the change in both groups is towards a darker coloration.

## Discussion

We here show the effect of background colour and temperature on colour changes and darkening in *G. sibiricus*. There were no colour morph switches among 78 individuals tested, neither when exposed to matched nor to unmatched background. Colour morph switches have been widely reported in other orthopterans (Table 1), hence it is interesting that we cannot confirm this for our species. If this species is capable of switching colour at all, the probability of colour morph switch in individuals that mismatched their cage background was certainly very low for both rounds of trials (≤7 %). Additionally, we find that the amount of radiant heat affected colouration darkening within nymphal stages, with darkening at low amounts of radiant heat and lightening at high amounts of radiant heat. Imagoes tended to darken within the first week after final ecdysis independent of radiant heat treatment, suggesting that imagoes may face different life-history trade-offs than nymphae.

### Green-brown switches

Colour morph switches have been previously reported, with several observations of the phenomenon in a diverse array of species (Table 1). Several of the species for which colour morph switches have been reported are members of the Acrididae (the family that also includes *Gomphocerus*), but none of them is a member of the subfamily Gomphocerinae [46]. It is possible that the capability for colour morph switches has been lost somewhere in the branch of Gomphocerinae, but this remains speculative in the absence of information about other species. The lack of a mechanism for switching colour during nymphal stages in *G. sibiricus* may also be due to the really fine-grained structure of the habitat inhabited by the species. It would be very costly for an individual to move at all within the matrix of colours of the habitat if this would require an active colour switching to match their background, even if this could be done in a relatively short time window.

Background colour was visually perceivable to the developing individuals and based on previous studies, we assume visual perception to be the main input that triggers colour change to match the background (see [14] and references therein). However, it might be argued that our coloured paper was not of the right kind for triggering colour changes. Previous studies have used a large diversity of materials for the background manipulation, such as stones, sawdust, sand, coal, clay, paper and paint (Table 1). These different materials have typically elicited colour morph switches, which suggests that the effect does not depend on the exact kind of materials used as background. We consider it unlikely (albeit possible) that our paper type was so substantially different from previously used materials that it would not be suitable for triggering colour switches.

In previous experiments, individuals have been tested for different periods of time in order to assess colour morph switches, usually starting the experiments at early nymphal stages [27, 32, 47–49]. Colour morph switches have typically been reported to occur across nymphal stages, often quantified a few days after ecdysis, though detailed information on the exact timing of switches is usually lacking. The only exception to colour morph changes occurring within nymphal stages are changes to black colouration, which have been reported to occur during the imaginal stage [14]. We started with our experimental treatment very early in the life of the grasshoppers (also in comparison with previous studies), giving scope for switches within and/or between developmental stages. Therefore it is rather unlikely that the duration of the treatment prevented colour switching.

Switches might also have been expected due to the radiant heat treatment, since brown individuals tend to be darker on average than green individuals and we would expect them to be better able to heat up if radiant heat is limiting. High temperature, as well as high humidity, high food moisture content and low individual density, are known to drive green body colouration in grasshoppers [14, 15]. We expect that in our experiment high radiant heat conditions would have served to cue individuals of a green habitat. High alpine habitats are typically characterized by high humidity regimes due to high condensation of air humidity at night, which is available as dew drops early in the mornings, and also due to high precipitation regimes [50]. Under these conditions, high temperature and high humidity will promote vegetation growth and produce greener habitats than conditions of low temperature, where vegetation would have weaker growth. Such conditions could have promoted colour morph switches from brown to green, possibly as a means for habitat matching. In contrast, low radiant heat conditions would have served to cue individuals of a browner habitat with less flourishing vegetation. These conditions, in the case of green individuals, could have promoted colour morph switches from green to brown, either for habitat matching and/or to improve thermoregulatory capacity.

Green individuals on a brown background and under the low radiant heat treatment thus constitute the subgroup for which colour morphs switches from green to brown appear particularly advantageous. It is possible that the combination of relative high humidity, high food moisture content and individual housing (i.e., low population density) counteracted the effect of the background, hampering the occurrence of the colour morph switch [14, 15]. This is different for brown individuals on a green background and under the high radiant heat treatment, since for those individuals the combination of low density, high humidity, background mismatching and no need for improved heat absorption are all expected to favour colour morph switches towards green. Yet none of the 16 individuals under this suit of conditions switched colour.

Our limited sample size does not allow us to exclude the possibility that *G. sibiricus* is capable of colour morph switches under some conditions. Still it strongly points against frequent, general developmental switches in response to background colour and temperature. We had expected that if colour morph switches occurred in *G. sibiricus*, they would occur at the nymphal stages, given that matching the habitat background is expected to improve survival in natural conditions. It is possible that nymphs of *G. sibiricus* achieve homochromy even in the absence of developmental switches by actively seeking out matching (micro)habitats [36]. Habitats of *G. sibiricus* are spatially highly heterogeneous and this might distinguish them from many of the other species that show developmental colour morph switches. Microhabitat variability might favour behavioural over developmental homochromy, while more global (temporal) variability in less structured habitats might favour developmental switches.

### Colouration darkening

While the temperature treatment in our experiment did not elicit colour morph switches, it elicited a more subtle response in colouration, causing darkening under low radiant and lightening under high radiant conditions. Radiant heat can limit behaviour of individuals by hampering thermoregulation and this in turn can constrain activity levels, growth and development, and ultimately fitness [51–53]. An increase in the amount of melanin under the cuticle surface would improve thermoregulatory capability due to a difference in heat absorbance between black and brown or green colours, and this would help counteract the effect of a short window of radiant heat exposure. Hence the darkening that we found in the low heat treatment is in line with what would be expected for improved thermoregulation [39–41].

The lightening in colouration in response to the high radiant heat treatment can result from a trade-off between melanin as a colour pigment and other functions of melanin. Melanin plays a role in several functions in insects, such as immune defence, integumental colouration, wound healing and cuticle sclerotisation, among others (reviewed in [54]). It has also been documented that melanin production in insects can be costly, mostly because of the many possible functions of melanin, but also because of dietary limitations of melanin precursors or lack of enzymes necessary to process precursors [51, 55–57]. Therefore lightening of colouration can be seen as an option to avoid investing melanin in body colouration when it is not necessary for absorbing more radiant heat. This reasoning might give an adaptive explanation for the lightening in our high radiant heat treatments.

A change in darkness within nymphal stages implies a mechanism which allows individuals to adjust the amount of visible melanin in their epidermis during the relatively short time spanned between moults. Such a mechanism would include cells at the epidermis capable of spreading pigment granules under the cuticle surface, but also capable of withdrawing the pigment granules under proper stimulation [19, 58]. Relatively little is known about the physiology of colour changes in grasshoppers, but different physiological and morphological mechanisms have been described in other arthropods.

A very intuitive mechanism which could explain the changes in darkness under different radiant heat regimes is pigment dispersal and concentration within chromatophores [19, 59]. This type of cell is known to be present in several taxa, such as fish, reptiles, amphibians, crustaceans and bacteria [60]. The shape of chromatophores is typically highly branched, allowing for pigment to disperse to the branches or contract to the centre to achieve colour change [19, 59, 61]. Another physiological mechanism involved in colour change is granule migration. Here granules of pigment are transported along microtubules which are perpendicular to the cuticular surface, and which branch distally, allowing the pigments to spread and therefore causing colour change. A striking example of this plastic mechanism is observed in the temperature-controlled daily changes in the colour of the chameleon grasshopper *Kosciuscola tristis*. In this case granules of pigment migrate from the epidermis when the grasshopper is exposed to temperatures above 25 °C, giving males a bright turquoise colouration [19]. A similar mechanism is used in the stick insect *Carausius morosus* [62]. The colour change that we found in *G. sibiricus* is much slower, but since granule migration might simultaneously explain changes in darkness in both directions, it might contribute to our observations.

The unpredictable climate in the habitat of *G. sibiricus*, characterized by long spans of time with few favourable climatic conditions, might explain the occurrence of a darkening and lightening mechanism. In this environment, given the duration of each nymphal stage (about one week, albeit likely to be substantially longer in the field) nymphae may need to adjust colouration even within nymphal stages to be able to cope with climatic variability. In species dwelling in habitats where radiant heat is a limited factor, energy balance and thus activity levels during early developmental stages could be hampered by limited sun exposure conditions. Being able to adjust colouration darkness could greatly improve the use of resources by an individual, in this case melanin which is a multipurpose and apparently costly pigment [63].

## Conclusions

Colour polymorphism is a striking feature of several species, which involves variation in the types of morphs and different environmental triggers and genetic determinants which combine to help define the colour morph of an individual [14, 38]. Here we report the absence of colour morph switches in the alpine grasshopper *G. sibiricus*. The lack of switches could be driven by the heterogeneity of the habitat of *G. sibiricus*. Additionally we found that nymphae of *G. sibiricus* are capable of modifying the amount of colouration darkness in response to radiant heat. This capability could help nymphae adjust colouration within nymphal stages according to the unstable climatic conditions characteristic of their habitat, and in turn improve allocation of pigment resources to other physiological processes. The complex net of interactions between fine-scaled variation in polymorphisms, unpredictable and highly variable environments, and melanin pathways which may be costly and also shared by different developmental processes are not only a very promising avenue of study in behavioural ecology, but they will also change the way in which we assess colour polymorphism in natural populations.

## Methods

### Subjects

Last-instar individuals of *G. sibiricus* were collected in the field (near Sierre, Valais, Switzerland) in July 2013 and brought to the laboratory where they moulted into imagoes. We housed imagoes in separate cages (dimensions 22 × 16 × 16 cm^3^) containing one male and one female and provided a cup of sand-vermiculite mixture as substrate for egg laying. Eggs were collected once per week, kept for approximately 6 weeks at room temperature and subsequently stored at 4 °C for diapause. After seven months in the refrigerator, eggs were taken out of their diapause and kept at room temperature until hatching. In total, 116 hatchlings hatched from 37 egg pods. Full-siblings from the same egg pod were housed together in the same cage (white cages lined with black mesh, dimensions as above) throughout the first nymphal stage, but were separated after the first nymphal stage (i.e., after about one week) and thereafter housed individually. We refer to the different nymphal stages as N2 (second nymphal stage), N3, N4 and IM (imago stage).

### Experimental setup

**Fig. 3:**
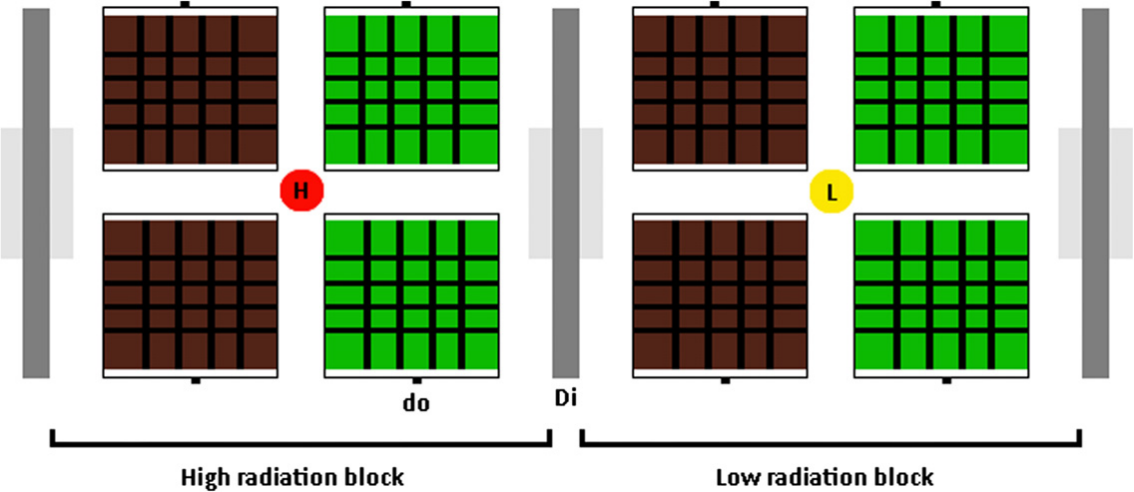
Experimental setup. Two blocks are shown, one exposed to the high radiant heat (orange dot) and one to the low radiant heat (yellow dot) treatment. Blocks are separated by dividers (Di). Each cage has its floor and two of its sides lined in green or brown paper, and each block has two cages lined with each colour. Time of exposure to radiant heat varied between rounds (see details in Temperature treatment section). The doors (do) on the sides allow easy access to cages

The experiments were conducted under artificial full-spectral light conditions (Biolux L 58 W/965, OSRAM, Munich, Germany) with a 14:10 H light dark cycle (7:00 till 21:00). Average humidity was kept at 70 % by humidifiers. In order to maintain a high moisture content in the food, we provided fresh grass in small plastic vials (height x diameter: 5.8 cm × 2.1 cm) filled with water, and replaced the grass every other day in order to avoid withered or yellowing grass. Experiments were conducted in two rounds (we refer to the two rounds as R1 and R2) that differed in details of the radiant heat treatment, because of high nymphal mortality under one of the conditions in R1. Experimental setups consisted of blocks of four cages (11 and 10 replicate blocks in the R1 and R2, respectively) flanked on both sides by isolating dividers (22 cm × 55 cm × 1 cm) (Fig. 3). The dividers were installed to isolate alternating blocks exposed to different experimental conditions. Individuals were transferred to experimental setups on the day of moulting into the N2 and assignment to treatments was done at random. All individuals remained under the same experimental conditions until one week after moulting into IM (unless they died before completing the experimental period). Only three individuals reached the last nymphal stage under low radiant heat conditions in R1, two of which died shortly after, and therefore the experiment was stopped at this point due to lack of one treatment group. We did not score the sex of individuals during early nymphal stages because this is easily visible during the N4 stage. In the case of the R1, individuals died rapidly while the experiment was running, and sex scoring was not assessed in time. For this reason data on colour morph frequencies for both sexes is not presented in the results.

### Background colour treatment

The first component of our experimental design involved an assessment of homochromy. Specifically we tested for changes in colour morph from green to brown or brown to green in response to differences in background colour. Each cage (same type as described above) was lined on two of its inner sides as well as the cage’s floor with coloured paper (Coloured drawing paper, green = #ADFF2F, brown = #8B4513, Folia Paper Bringmann, Wendelstein, Germany). Each experimental block consisted of two cages lined with brown colour and two cages lined with green colour. Individuals had visual contact to only one neighbouring cage that was ensured to be of the same colour as their own cages (Fig. 3).

### Temperature treatment

The second component of our experimental setup consisted of a temperature treatment and was applied to entire blocks. Grasshoppers regulate their body temperature behaviourally, elevating it substantially above ambient temperature by sun-bathing [24]. We aimed to simulate high (sunny) and low (overcast) radiant heat conditions, both of which occur in the natural habitats of *G. sibiricus*. Therefore, we placed 150 W infra-red heat bulbs (IOT/90, Elstein, Northeim, Germany) at the vortex of each block. In the R1, blocks were exposed to either 5 h (high treatment) or 1 h (low treatment) of radiant heat, but due to high mortality, this was increased to 4 + 6 h (high treatment) or 2 h (low treatment) per day in the R2 (Fig. 3). The hours of exposure in the R1 for the high treatment were from 9:00 until 12:00, and again from 13:00 until 15:00, while the exposure time for the low treatment was from 12:00 until 13:00. The hours of exposure in the R2 for the high treatment were from 8:00 until 12:00, and again from 14:00 until 20:00, while the exposure time for the low treatment was from 12:00 until 14:00. The temperature at exposed locations was around 44 °C when heat lamps were active, in contrast to the 25 °C when heat lamps were switched off. In the high radiant heat treatment, individuals were able to adjust the amount of radiant heat that they received by positing themselves at the mesh ceiling or by moving away from it or also by hiding behind the beams of the cage.

### Scoring of body colour

Colour morphs are identifiable from the third day of the second nymphal stage. Both morphs vary in the degree of colouration darkness (e.g., from very light green, across a discreet range of green tones, to very dark green morphs). Pale versions (which presumably have reduced bile pigments, ommochromes and melanin – responsible for green, brown and black colouration, respectively; [6, 35]) as well as melanic versions (very dark in colouration) occur in the lab and in the field. We defined two intermediate categories among those extremes, based on a collection of photographs of individuals taken either in the lab or in the field on previous years. The scoring was done every third day, assigning the individuals a colour grade based on the degree of darkness in the area under study. The categories where defined as follows: 1. Very light = < 10 % of black; 2. Light = 30-40 % of black; 3. Dark = 60-70 % of black; 4. Very dark = > 90 % of black. Our analysis is based on a total of 433 scorings from the R1 and 306 scorings from the R2.

### Statistical analysis

Since our sample size is necessarily finite, it is at least possible that despite an absence in our sample, colour morph switches occur with a low probability. We therefore estimated the lowest probability of colour morph switches that would still be consistent with our data. We used the following approach to assess the highest colour change probability that is consistent with our data. We define *c* as the probability of colour change in unmatched backgrounds, (1*-c*) as the probability of no change and *k* as the number of individuals exposed to unmatched backgrounds. Based on binomial sampling, we expect to observe no colour morph switches among *k* individuals with a probability of (1-*c*)^k^. We defined a probability threshold of α = 0.05 and searched for the value of *c* at which the expectation of no-colour morph switches is equal to α. This gives the highest possible switching probability that is still considered reasonably consistent with our data.

To test for the effect of the temperature treatment in the body colour we used a random-slope mixed effects model. We chose this model because the possible response to the treatment could vary physiologically from individual to individual, making it necessary to account for this variability in our analysis [64]. The p-values for the fixed effects in the mixed models were approximated by a t distribution with degrees of freedom equal to the number of individuals minus the number of fixed effect parameters estimated in the model. This approach is conservative in that it considers only individuals as independent datapoints. However, such random-slope models depend on assumptions about the linearity of the response-covariance relationship and about the distributions of random-deviations from the population mean and population slope. Therefore, we applied additional, simpler tests for the same question in order to verify the robustness of the results (see below for details).

In addition to the analysis of the data using random slope models, we considered using alternative ways of analysis to confirm the robustness of the results on colouration darkening. First, we identified those individuals who showed an unambiguous lightening or darkening in colour (individuals with bidirectional changes in colour darkening within a nymphal or imago stage were left out: R1, *n* = 10 nymphae + 1 imago; R2, *n* = 2 imagoes). Then we counted the number of cases of darkening and lightening in each stage, and compared them using Fisher’s exact tests. Second, we compared treatments per observation time (early or late observations within nymphal stages) between treatments using two sample t-tests. Third, we compared the darkness between the earliest and latest observations per treatment within nymphal stages using paired t-tests. This was only possible for those individuals that had at least two observations per nymphal stage. All analysis were conducted in R 3.1.1 [65], and we used the lme4 package [66] for mixed model fitting.

## Availability of supporting data

The data sets supporting the results of this article are available in the Dryad repository: doi:10.5061/dryad.j95t2 [67].

## Abbreviations

N1: First nymphal stage
N2: Second nymphal stage
N3: Third nymphal stage
N4: Fourth nymphal stage
IM: Imaginal stage
R1: First round
R2: Second round
